# Progress in Medicine

**Published:** 1900-05-05

**Authors:** 


### Progress in Medicine.

### RHEUMATISM AND GOUT.

Westphal and his colleagues[1] claim that they have isolated a micrococcus in acute rheumatism, which, when cultivated in an alkaline medium, and then injected into animals, produces fever with acute multiple arthritis. The organism appears to be a streptococcus, though at times it takes the form of a diplococcus. The patient in whom it was found is said to have suffered from acute rheumatism, followed by chorea, with endocarditis and nephritis, a list of complaints that raises a suspicion, however, of some form of septicæmia. The animals, too, who were infected by it usually died after the joint affections were established. While the ordinary endocarditis of acute rheumatism is of a non-malignant type, cases have been recorded by Bristowe, Latham, and others of a true malignant form appearing in simple rheumatic fever. E. Barié[2] reports two cases of this kind, and discusses the cause of the severity of the symptoms. He considers that the result may be due either to the failure of the myocardium and consequent dilatation, to special susceptibility of the patient, to intensity of the rheumatic poison, or to the entrance of some secondary infection. In a discussion on the relation of chorea to rheumatism, E. D. Fisher[3] insisted on the rarity of rheumatic and cardiac symptoms in chorea, apart from irregular action of the heart; and the success of its treatment by iron, cod-liver oil, and arsenic, which have little effect in rheumatism; while finally the age incidence of the two diseases differs, chorea being more frequent before 15, and rheumatism afterwards. F. M. Crandall, on the other hand, held that the symptoms in children often commence with chorea, and only show joint or heart affections in later years. In 130 chosen cases of his own there were nearly half in which he noted previous or concurrent rheumatic symptoms, and many in which these appeared in later years.

**Treatment.**—F. H. Wiggin praises[4] salol in place of the salicylates in acute rheumatism. He gives 120 or 160 grains in divided doses during the first 24 hours, and then smaller quantities to prevent a relapse. Indeed, he finds a combination of acetanilide with salol preferable to any other remedy. A. A. Smith also advises the use of salol in the acute stage in doses sufficient to affect the urine. Aspirin is praised by Flöckinger[5] for acute and chronic rheumatism. It is only absorbed from the intestine, and does not irritate the stomach, depress the heart's action, or cause tinnitus in ordinary doses. The writer took 135 grains himself in two hours, but gives the usual dose as 45 grains per diem. The hot air treatment is highly praised by J. R. Willis, of Melbourne,[6] who describes its results in 10 cases of various joint affections. Five were cured, and five markedly relieved. S. S. Dunn,[7] of Adelaide, has also recorded the results of 40 cases. He notices the absence of any bad effect on the heart, even where valvular disease is present, the immediate relief of pain, and the reduction of swelling. In gout he thinks that permanent good is done, but in rheumatoid arthritis though great temporary relief is given, relapses occur unless a very prolonged course of baths is taken. For sciatica he combines the baths with painting H.Cl. over the painful area. On the whole the treatment is a valuable aid in a difficult class of cases. Whitby[9] suggests that it is better to employ a hot-air bath for the whole body, for in rheumatic and gouty cases a local one will often shift the inflammation from one joint to another. He finds that luminous heat baths are the most powerful diaphoretics in existence, and are also very valuable in some skin troubles. He prefers them to any other form of hot bath for gout, sciatica, and rheumatoid arthritis. For the last mentioned, however, probably the best of all remedies is the continuous, or sinusoidal, current bath, in which very excellent results have been attained.

C. G. Skinner[9] reports most favourably of "local" hot-air treatment. He regards the theory that it may cause a metastasis of the inflammation as absurd, and claims that it has no equal for the relief of pain, though in the case of rheumatism this relief is only temporary. It is of the greatest importance to continue ordinary medicinal treatment during the course of hot-air baths, since much better, and far more certain effects are thus obtained than when either treatment is employed alone. In this sense the hot air has a permanently curative value. The shortening of the duration of pain is very remarkable, and he gets equally good results in the treatment of pleurisy and abdominal pain. It is of importance to watch the patient during a bath, and to adjust the valves if he complains of any burning. Should the porous cloths fail to touch the skin so as to absorb and withdraw from it the sweat, boiling quickly takes place, and the skin is blistered. Accidents are, however, very rare, and the writer has even employed a temperature of 500 deg. Another paper of great interest is that by H. Lewis Jones,[10] describing in detail the electrical treatment employed in his department at St. Bartholomew's Hospital. Here the sinusoidal current from the street mains is largely used in the form of general and local electric baths, and, except where acute pain is present, its results are more satisfactory than those of the continuous current. He, like Dr. Whitby, finds the sinusoidal current applied by a bath to the whole body the best of all treatment for rheumatoid arthritis. This form of current is important, because in most large towns it is so easily obtained and worked at the present time, but it does not seem to lend itself to direct application by electrodes to the body on account of the unpleasant sensation it produces. Hence the greatly increased use of arm, leg, and general baths, in which the current is distributed by water to the skin in a mild and yet thoroughly efficacious form. As the writer notices, there is also the incidental advantage that these baths are much less trouble to give than the ordinary application of electricity by electrodes. The current is simply turned on and the patient immerses the limb in the water, taking care not to touch the electrodes, and keeps it there for the desired time, perhaps 10 or 15 minutes.

**Allied Disorders.**—Arthritis deformans was formerly thought to be connected with gout, and Garrod finds a history of some joint affections in nearly 50 per cent. of his patients; but instances where the disease itself has appeared in relations of a patient have been very rarely recorded. J. S. Billings[11] describes a case where the mother and grandmother were both affected at about the same age as the patient, who suffered for twelve years from typical rheumatoid arthritis. A discussion in the New York Neurological Society took place upon the varieties of stiff spine. In some of the cases described by Strümpell and Marie there is a resemblance to rheumatoid arthritis, but in others Von Bechtereff has found a meningitis leading to degeneration of certain spinal roots, and to corresponding symptoms in the nerve centres supplied by them. Dana[2] thinks that many of the former are merely instances of the senile type of arthritis deformans, affecting either the spine or the hips or both together. G. R. Elliott holds that they are due to a proliferating arthritis and not to arthritis deformans, though it must be allowed that the small joints are sometimes affected. Much difference of opinion, however, seems to exist as to the type of disease to which this spondylosis rhizomélique, as Marie named it, really belongs.

[1] Med. Rec., Nov. 18. [2] La Semaine Med., Jan. 31. [3] New York Med. Jour., Jan. 20. [4] Ibid. [5] Brit. Med. Jour., Dec. 9. [6] Australasia Med. Gaz., Aug. 21. [7] Australasia Med. Gaz., Nov. 20. [3] Bristol Med. Chir. Jour., Mar. [9] New York Med. Jour., Oct , Dec. [10] Practitioner, Sept. [11] Med. Rec., Sept. 30. [14] New York Med. Jour., Nov. 25.

